# Excretion of Transmissible Spongiform Encephalopathy Infectivity in Urine

**DOI:** 10.3201/eid1409.080259

**Published:** 2008-09

**Authors:** Luisa Gregori, Gabor G. Kovacs, Irina Alexeeva, Herbert Budka, Robert G. Rohwer

**Affiliations:** Veterans Affairs Medical Center, Baltimore, Maryland, USA (L. Gregori, I. Alexeeva, R.G. Rohwer); University of Maryland, Baltimore (L. Gregori, R.G. Rohwer); Medical University of Vienna, Vienna, Austria (G.G. Kovacs, H. Budka)

**Keywords:** Prion, urine, TSE, kidney, bladder, endogenous infectivity, scrapie, hamster, limiting dilution titration, pathology, research

## Abstract

The route of transmission of most naturally acquired transmissible spongiform encephalopathy (TSE) infections remains speculative. To investigate urine as a potential source of TSE exposure, we used a sensitive method for detection and quantitation of TSE infectivity. Pooled urine collected from 22 hamsters showing clinical signs of 263K scrapie contained 3.8 ± 0.9 infectious doses/mL of infectivity. Titration of homogenates of kidneys and urinary bladders from the same animals gave concentrations 20,000-fold greater. Histologic and immunohistochemical examination of these same tissues showed no indications of inflammatory or other pathologic changes except for occasional deposits of disease-associated prion protein in kidneys. Although the source of TSE infectivity in urine remains unresolved, these results establish that TSE infectivity is excreted in urine and may thereby play a role in the horizontal transmission of natural TSEs. The results also indicate potential risk for TSE transmission from human urine–derived hormones and other medicines.

## Abstract 2

Infectivity in hamster urine indicates a possible route of horizontal transmission of natural sheep scrapie and poses a potential risk in human urine-derived pharmaceuticals.

Transmissible spongiform encephalopathies (TSEs) are fatal neurologic diseases. In humans, a long asymptomatic incubation period is followed by a progressive clinical course that typically lasts a few months to a year. TSE infectivity and pathologic changes are concentrated in the nervous system; however, much of the transmission risk results from parenteral exposure to the much lower concentrations of infectivity found in tissues outside the nervous system. Thus, despite the very low concentration of TSE infectivity in blood (*1*,*2*), 4 human cases of transmission of variant Creutzfeldt-Jakob disease through blood transfusions have been documented (*3*,*4*). If TSE infectivity were excreted, human urine, which is a source of injectible fertility hormones and other drugs (*5*,*6*), could also pose a risk for transmission. Infected urine might also account for the horizontal transmission of sheep scrapie and might contribute to the natural spread of chronic wasting disease in deer and elk.

Early attempts to transmit Creutzfeldt-Jakob disease by cross-species inoculation of rodents and primates with urine from diseased patients failed (*7*,*8*). More recent attempts in which urine from infected hamsters was injected back into hamsters have produced variable results (*9*,*10*). Two other studies have reported infectivity in urine (*11*) and infectivity with disease-specific prion protein (PrP^d^) in kidneys of mice with simultaneous scrapie and nephritis but not in those with scrapie alone (*12*). To resolve these discrepancies, we used a highly sensitive and precise method of measuring low concentrations of TSE infectivity, which we have successfully used for quantitation of TSE infectivity in blood (*1*,*2*), to measure the concentration of TSE infectivity in urine of scrapie-infected hamsters.

## Materials and Methods

### Urine Collection and Processing

Urine was collected from a cohort of 22 Syrian hamsters (Harlan Sprague-Dawley, Haslet, MI, USA) that had been infected by intracranial injection with 10% (wt/vol) scrapie brain homogenate (263K strain) and from a cohort of 8 age-matched, noninoculated control animals. At the time of urine collection, the scrapie-infected hamsters showed clear clinical evidence of disease but were still able to drink and eat (67–74 days postinoculation). Hamsters were placed 2 at a time for 24 hours in metabolism cages in which they had access to water but not food. Food was withheld to prevent contamination of the urine. Urine was maintained at 4^o^C during collection. Separate metabolic cages (Rat metabolic cage no. 2100-R; Lab Products, Seaford, DE, USA) were used for each cohort. The urine produced daily was stored at –80^o^C. The individual collections were then combined into clinical and control pools of ≈60 mL and ≈125 mL, respectively.

### Limiting Dilution Titration of Urine

We used the limiting dilution method of titration developed in our laboratory to measure the concentration of TSE infectivity in urine (*1*,*2*). In this method, a relatively large volume of low-titer sample is injected intracerebrally, 50 μL at a time, into a large cohort of weanling hamsters. Immediately before animal inoculation, aliquots of the clinical and control urine pools were thawed and sonicated on ice with separate sterile ultrasonication probes for each pool. Sonication was for 4 cycles of 15 s on and 10 s off for 1 min of total sonication, using a microtip probe at 40% amplitude (Vibra-Cell 750W; Sonics & Materials, Newtown, CT, USA). Two milliliters of control urine was injected undiluted into 40 hamsters. Clinical urine (urine from hamsters showing clinical signs of disease) was diluted 1:3 with inoculation buffer (phosphate-buffered saline [PBS] supplemented with 1% fetal calf serum and 1× penicillin and streptomycin) to remove concentration-related toxicity. Five milliliters from the clinical urine pool was diluted to 15 mL, and the entire volume was injected into 300 hamsters, 50 μL/animal. Soon after inoculation, 8 animals inoculated with urine from the infected animals died, which left 292 animals in the study. All inoculations were conducted under anesthesia with pentobarbital (40–90 mg/kg). At each step the control urine was processed before the infected urine.

All animals were assessed weekly for early signs of scrapie. At the first signs of disease, animals were separated from their cage mates, observed daily for disease progression, and euthanized after disease was confirmed clinically. After 559 days postinoculation all remaining animals were euthanized. Brains were collected from all animals in the study and assayed for infection-specific, proteinase K–resistant prion protein (PrP^res^) by Western blot or ELISA, using a dissociation-enhanced lanthanide fluorescent immunoassay (DELFIA) developed in our laboratory (described below). The infection status of each animal was tabulated, and the probabilities of infection and titer were computed as described (*1,2*; Table 1).

**Table 1:** Titer of urine from scrapie-infected hamsters

Hamster	Volume assayed, mL	Fold dilution	Volume inoculated, mL	Total no. hamsters	No. infected hamsters	Titer, ID/mL*	SD†
Infected	4.87	3	14.6	292	18	3.8	0.9
Noninoculated	2	None	2	40	1	–	–

*ID, infectious dose. Titer = –ln(P(0)) × (1/v), where P(0) = (noninfected animals)/(total animals inoculated) and v = inoculation volume, 0.05 mL. †SD = square root (titer/V), where V = 4.87 mL, the total volume of the undiluted urine inoculated (*1*).

### Tissue Collection and Processing

Kidneys and urinary bladders were harvested from each of 12 infected animals that donated urine either 71 or 76 days postinoculation. Animals were euthanized by asphyxiation with CO_2_. The bladder was removed first and immediately frozen in liquid nitrogen. The kidneys were collected next; the renal capsule was removed before freezing the tissue in liquid nitrogen. Both tissues were dissected aseptically with a clean, sterile set of instruments for each animal and each organ; particular care was taken to not touch other organs or tissues. The tissues (12 bladders and 19 kidneys) were pulverized with a cryomill by using separate cryo-capsules for each tissue (Cryogenic Sample Crusher, Model JFC-300; JAI, Tokyo, Japan). The tissue powder was stored at –80^o^C until use.

### End-Point Dilution Titration of Tissues

Pooled bladder powder (1.65 g) and pooled kidney powder (0.64 g) were separately mixed with homogenization buffer (PBS, pH 7.2) to make 10% (wt/vol) tissue suspensions before sonication at 40% amplitude, using separate sterile microtip probes for each homogenate. The kidney homogenate was prepared according to the same schedule of sonication used for the urine pools. The bladder homogenate was sonicated for 10 s, repeated 2 times (20 s total sonication time) at room temperature. Longer sonication times or delays in the injection of the bladder homogenate caused the sample to solidify, which made it impossible to dilute and inject. Immediately after sonication the homogenates were serially diluted 10-fold in inoculation buffer, and each dilution was injected into hamsters in 1 to 5 cages (4 hamsters/cage) for titration by end-point dilution (Table 2).

**Table 2:** End-point dilution titration of urinary bladder and kidney from scrapie-infected hamsters

Dilution	Total/no. infected	
	Bladder	Kidney
10^–1^	19/19	4/4
10^–1.3^	8/8	20/20
10^–1.7^	8/8	8/8
10^–2^	4/4	8/8
10^–3^	4/4	4/3
10^–4^	4/2	4/1
10^–5^	4/1	4/0
10^–6^	4/0	4/0
Titer (log_10_ ID_50_/g)*	5.5	5.0
Standard error	0.5	0.4

*ID_50_, 50% infectious dose. Titers calculated by the Reed and Muench method (*13*); standard errors by the Pizzi method (*14*).

All dilutions were by weight. The study was terminated at 426 days postinoculation, and the infection status of each animal was confirmed by Western blot of the brain for PrP^res^. The titers were calculated by the methods of Reed and Muench (*13*), Pizzi (*14*), and Spearman and Karber (*15*).

### PrP^res^ Detection Procedures

### Immunoblotting

Individual brains were homogenized in PBS, pH 7.2, to 10% (wt/vol) by using a FASTH homogenizer (Consul AR; Villenueve, Switzerland) according to the manufacturer’s instructions. To test for PrP^res^, brain homogenate was digested with proteinase K at 0.1 mg/mL final concentration as described by Gregori et al. (*1*). Sample buffer containing 2% sodium dodecyl sulfate was added, and the samples were heated at 100^o^C for 10 min and subjected to sodium dodecyl sulfate–polyacrylamide gel electrophoresis. Western blots of the samples were developed by using anti-PrP 3F4 monoclonal antibody (Covance, San Diego, CA, USA) for PrP detection (*1*).

### ELISA

After proteinase K digestion and heat denaturation as described for immunoblotting, the samples were diluted 100-fold in assay buffer (DELFIA Assay Buffer; PerkinElmer, Waltham, MA, USA). They were then assayed for PrP concentration by DELFIA by using a Wallac Victor V instrument (PelkinElmer) for signal detection, with purified 3F4 monoclonal antibody (Covance) as the capture antibody and purified 7D9 monoclonal antibody (Covance) labeled with Europium according to the manufacturer’s instructions (PerkinElmer) as the detection antibody. The molar ratio of Europium:7D9 antibody was 7.4:1 (*2*).

### Histologic and Immunologic Tissue Preparation

Formalin-fixed brains were cut and divided on the midline; 1 hemisphere was cut in the sagittal plane; the other was cut coronally at the anterior basal ganglia, the middle of the thalamus, and the brainstem with cerebellum. Spleens, kidneys, and bladders were divided in the middle. All blocks were embedded in paraffin and processed for conventional staining with hematoxylin and eosin and Luxol fast blue/nuclear fast red (for brain) as well as for immunohistochemical detection of PrP with monoclonal anti-PrP antibody 3F4 (1:1,000; Covance). For detection of PrP^d^, sections were pretreated with 30 min of hydrated autoclaving at 121°C followed by 5 min in 96% formic acid. Immunostained sections were counterstained with hematoxylin.

### Animal Husbandry and Decontamination Procedures

Animals were maintained in a Biosafety Level 3 (BSL-3) animal facility at the Veterans Affairs Medical Center in Baltimore, Maryland, USA. Standard operating procedures specifically designed for TSEs, including TSE select agents, were followed. The operation of this facility has been described in detail (*16*). Animal cages were changed once a week, and cages and bedding were decontaminated by autoclaving for 1 h at 134^o^C. The sonicator probes and dissection instruments were decontaminated by autoclaving for 2 h at 134^o^C immersed in 2 N NaOH, followed by cleaning, repackaging, and sterilizing. All laboratory surfaces were decontaminated before use with either 2 N NaOH or LpH (Steris Corporation, Mentor, OH, USA) (*16*).

## Results

### Urine Titration

Urine collections from infected and control animals were combined into separate pools. Pools minimized the possibility of an idiosyncratic measurement from an individual and serve as a resource for future experiments once the titer has been determined. Clinically affected animals consumed lower amounts of water and produced 4–5-fold less urine than control animals. This resulted in slightly elevated specific gravity, proteins, glucose, and ketones as measured with a standard urine dipstick. Elevated urine ketones may also have been caused by fasting. The higher concentration of the urine pooled from infected animals resulted in a toxicity that required a 3-fold dilution in buffer before it could be injected.

**Figure 1:**
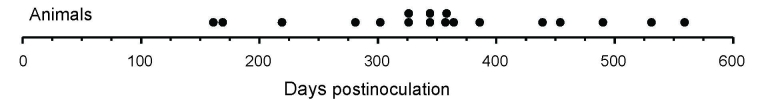
Distribution of incubation times of hamsters infected by injected urine. Each dot represents 1 animal with clinical scrapie that was euthanized at the corresponding day postinoculation. The 22 additional animals that died during the incubation period and the 252 animals that survived to the end of the experiment (559 days) showed no clinical or immunochemical evidence of scrapie and were scored as scrapie negative.

TSE developed in 18 of the 292 animals that survived the injection of the 3-fold diluted infected pool. Incubation times are shown in Figure 1. As observed in other studies (*1*,*2*), scrapie incubation times for animals infected with low-titer samples begin at ≈150 days and rarely extend past 500 days. None of the animals from either the infected or noninfected cohorts that survived to the end of the experiment were positive by DELFIA. None of the 24 animals that died during incubation without clinical evidence of scrapie were positive for scrapie infection by Western blot. Only those animals with clinical scrapie had the typical PrP^res^ signal in the brain as assessed by Western blot. The infectivity titer of the urine as calculated from the Poisson distribution was 3.8 ± 0.9 infectious doses (ID)/mL (Table 1).

Scrapie developed (at 425 days postinoculation) in 1 of the 40 hamsters inoculated with control urine. Because none of the control donor animals contracted scrapie and because their brains were negative for PrP^res^, it is clear that this infection resulted from contamination. However, the contamination was unlikely to have been environmental. Our BSL-3 is managed under a strict regimen of continuous decontamination and precautionary cleaning (*16*). As evidence of the effectiveness of these measures, we have conducted several titrations, involving hundreds of animals each, in which there were no infections at all during >540 days of incubation. One such study was ongoing during the titration of the urine pools reported in this study (*2*). If there are environmental sources of infectivity, the concentration is below the level of detection by the data accumulated in infection-free titrations to date. Instead, after an intensive review of our procedures, we concluded that the most likely source of this contamination was a technical lapse during collection of the urine pools. The level of contamination (1 infection/2 mL of control pool injected vs. 18 infections/4.87 mL of clinical pool injected) is consistent with a pooling error at the time of collection. Nevertheless, had it been an environmental contamination, the associated titer (0.51 ID/mL SD = 0.50 ID/mL) would have had a negligible effect on the value determined for the infected urine.

### Tissue Titrations

The concentration of scrapie infectivity in hamster urine is similar to that in plasma of scrapie-infected hamsters at the same stage of disease, which suggests plasma as a possible source of the infectivity. To investigate other possible sources, we also measured the concentration of TSE infectivity in separate pools of kidneys and bladders collected from the same donor animals. The titrations were by the end-point dilution method. The titers calculated by the methods of Reed and Muench (*13*) and Pizzi (*14*) were 10^5.5 ± 0.5^ 50% infectious doses (ID_50_)/g of bladder and 10^5.0 ± 0.4^ ID_50_/g of kidney. The Spearman and Karber method gave almost identical values (*15*).

### Histologic and Immunohistochemical Examination of Tissues

**Figure 2:**
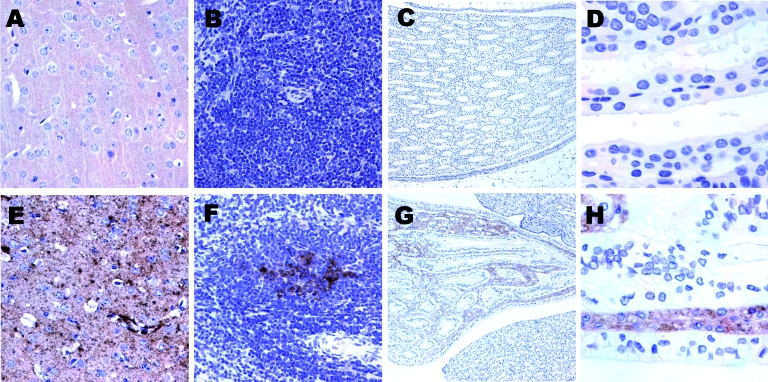
Immunostaining for prion protein (PrP) in control and scrapie-infected hamsters. Deposition of disease-associated PrP is lacking in the brain (A), spleen (B), and kidneys (C,D) of control hamsters. Fine synaptic and plaque-like PrP immunoreactivity in the frontal cortex (E), granular immunoreactivity in the germinal center of spleen (F) and in the collecting tubules of kidneys (G,H) in a representative scrapie-infected animal. Original magnification ×200 for panels A, B, D, E, F, and H and ×40 for panels C and G.

Others have reported TSE infectivity in the urine of scrapie-infected mice with nephritis but not in infected mice without nephritis (*11*,*12*). In contrast, our hamster colony in general, and the animals in this experiment, showed no evidence of inflammation, as indicated by clinical assessments or urine parameters. Nitrates were within normal limits, and no leukocyturia was noted. Proteinuria in the clinical hamsters was likely the consequence of low-volume urine excretion. To further assess whether hamsters infected with scrapie were also affected by kidney inflammation or other abnormalities of the urinary system, we examined the kidneys and the urinary bladders of 8 scrapie-affected hamsters at 84 days postinoculation and 4 preclinically infected hamsters at 49 days postinoculation for PrP^d^ by immunohistochemical and histologic methods (Figure 2). We also examined control tissues from 10 age-matched uninoculated animals as well as brain and spleen tissues from infected and control animals.

All tissues were evaluated for signs of inflammation and for the pattern of PrP^d^ immunoreactivity; brains were also examined for spongiform change. No inflammatory changes were found in any tissue examined. In 9 infected animals (clinical and preclinical), we noted nidus formation in the lumina of the bladder with a few neutrophilic granulocytes. However, leukocytes had not invaded the wall of the bladder. Nidus formation is often associated with dehydration.

PrP immunoreactivity was not observed in the bladder wall of scrapie-infected or control animals (data not shown). Spongiform change and deposition of PrP^d^ was lacking in control animal brains (Figure 2, panel **A**) and was noted to various extents, according to the stage of the disease, in all scrapie-infected animal brains (Figure 2, panel** E**). We observed fine synaptic PrP^d^ immunoreactivity with focal patchy or plaque-like appearance in gray matter structures, but we also noted ependymal, subependymal, perivascular, and white matter PrP^d^ deposits (data not shown). PrP^d^ immunoreactivity was observed in the germinal centers of the spleen of all scrapie-infected animals (Figure 2, panel **F**) but not in those of controls (Figure 2, panel **B**). None of the control animals exhibited immunoreactivity for PrP^d^ in the kidneys (Figure 2, panels **C**, **D**). PrP^d^ immunostaining showed fine granular deposits in the collecting tubules of the medulla (Figure 2, panels **G**, **H**) in 4 (50%) of 8 animals in the clinical stage of scrapie and in 3 (75%) of 4 animals in the preclinical stage, for a total of 7 (58.3%) of 12 scrapie-infected animals.

## Discussion

Anticipating that the titer of scrapie infectivity in excreted urine would be low, we measured concentration by using limiting dilution titration, a method with which we have extensive experience quantitating TSE infectivity in blood and blood components. In a limiting dilution titration, all animals in the bioassay are inoculated with the highest concentration of inoculum that is tolerated by the intracranial (most efficient) route. Infectivity assorts randomly into the inoculated animals; provided that at least some, but not all, of the animals are infected, the concentration can be calculated from the Poisson distribution of the infections (*1*). The method is highly sensitive and far more precise than other methods of TSE titration. We considered concentrating the urine before bioassay, but to circumvent uncertainties about the recovery of endogenous infectivity, we decided to inject the urine as collected.

We found TSE infectivity in the urine of hamsters that had no evidence of kidney or bladder inflammation. In contrast, Seeger et al. did not detect infectivity in the urine of scrapie-infected mice (*11*) unless the mice were also affected by nephritis, in which case they found low levels of infectivity. Whether the bioassay they used was capable of detecting infectivity at the concentration we observed for hamsters is not clear. If it was not capable, then detection of infectivity in mice with nephritis implies a higher concentration of infectivity in urine excreted by a nephritic kidney. In another study, urine and feces from deer with chronic wasting disease failed to demonstrate infectivity when orally given to the same susceptible species (*17*). Although usually an inefficient route of inoculation, the oral route did successfully transmit chronic wasting disease infectivity in saliva. The authors identified several possible reasons for the unsuccessful transmission by excreta, including incubation time, genotype, or sample size.

In our experiments, cross-contamination by feces can not be excluded as a source of infectivity. Although the metabolism cage effectively separated urine and feces, some contact is possible because of the anatomy of the hamster.

Protein misfolding cyclic amplification uses sonication to generate PrP^res^ and infectivity in vitro. Although we routinely disperse all samples by ultrasonication before injection, our conditions are much harsher than those used to generate PrP^res^ de novo (*18*) and do not support protein misfolding cyclic amplification of PrP^res^, or presumably infectivity (L. Gregori and R.G. Rohwer, unpub. data).

The kidney and bladder titers were far greater than expected compared with findings of historical studies in which, with only rare exceptions (*19*–*21*), most attempts at transmission have been unsuccessful. These titers cannot be explained by the infectivity in residual blood (10 ID/mL) (*1*,*2*). In addition, we observed PrP^d^ in the kidneys of scrapie-infected animals that had no indications of tissue inflammation. Heikenwalder et al. found PrP^d^ staining within follicular infiltrates only in kidneys of mice affected by nephritis and not in control mice with noncomplicated scrapie (*12*). These data together with those by Seeger et al. (*11*) suggested that renal inflammation might be a prerequisite for TSE infectivity in renal tissue and its excretion in urine. In contrast, our results indicate that renal inflammation is not necessary for the deposition of PrP^d^ in kidneys or for excretion of infectivity. One interpretation is that nephritis enhances the accumulation of PrP^d^ at sites of inflammation, consistent with the excretion of higher levels of infectivity inferred above for this same condition (*11*).

Two studies of scrapie in naturally and experimentally infected sheep reported PrP^d^ depositions in the renal papillae (*22*) and in the intraepithelial cortex, medulla, and papillae (*23*). Similar to our findings, both studies indicated that not all scrapie tissues examined were positive for PrP^d^. In chronic wasting disease, PrP^d^ staining was uniquely localized in the ectopic lymphoid follicle of the kidney of a whitetail deer (*24*). All studies indicated either no changes (*22*,*24*) or mild to no inflammatory changes of the kidney (*23*). Thus, our histologic and immunohistochemical results for scrapie-infected hamsters are consistent with results found for sheep and deer and suggest that under normal conditions TSE diseases do not have concomitant inflammatory changes in the kidney.

That urine titer is similar to that of plasma suggests that urine infectivity may originate from blood (*25*), but how the infectivity would be excreted is not clear. In general, proteins >40 kDa are not excreted and smaller proteins crossing the glomeruli are reabsorbed in the renal tubule and returned to the blood. If TSE infectivity is particulate (>40 kDa), its presence in urine might indicate abnormalities in renal filtration, perhaps related to the accumulation of PrP^d^ in the collecting tubules of the medulla. The accumulation of immunoglobulins in the urine of TSE-infected hamsters and humans may also indicate malfunction of the urinary system (*9*,*26*). Excretion of a small C-terminal fragment of the normal cellular form of the prion protein in urine of infected and noninfected animals has been reported (*27*), but PrP^res^ or PrP^d^ forms can only be inferred from the presence of infectivity. Nevertheless, excretion of proteins similar to PrP^res^ or PrP^d^ forms has been documented. Follicle-stimulating hormone is a glycosylated protein of 203 amino acids organized mostly as a β-sheet, which bears some remarkable similarities to β-rich forms of the prion protein. Follicle-stimulating and several similar hormones are excreted in urine at great enough concentration to be extracted commercially. Alternatively, TSE infectivity may be excreted by processes analogous to those responsible for the low-level virurias that occur during infections of the nervous system by mumps, measles, and West Nile virus (*28*–*30*).

To the extent that results from the hamster model can be generalized to other TSE infections (and it has so far proven highly predictive), then even the very low concentrations of infectivity measured here could result in substantial environmental contamination. Several liters of urine and several thousand doses of TSE infectivity may be excreted daily over the course of the illness; even higher titers might be excreted by an animal with nephritis. The high stability of TSE infectivity would account for its persistence in pasture years after infected animals are removed (*31*). Recent studies have shown that infectivity that is adsorbed and immobilized by soil minerals (*32*) can still infect hamsters by oral exposure 29 months later (*33*). Our study also warns of a possible risk from TSE contamination to fertility hormones and other medicinal products extracted from human urine.
